# Effect of Micronization on *Panax notoginseng*: *In Vitro* Dissolution and *In Vivo* Bioavailability Evaluations

**DOI:** 10.1155/2021/8831583

**Published:** 2021-01-18

**Authors:** Xiao Liang, Guobing Xu, Zhenbao Li, Zihua Xuan, Hongsu Zhao, Daiyin Peng, Shuangying Gui

**Affiliations:** ^1^Anhui University of Chinese Medicine, Hefei, China; ^2^Anhui Institute for Food and Drug Control, Hefei, China; ^3^Institute of Pharmaceutics, Anhui Academy of Chinese Medicine, Hefei, China; ^4^Key Laboratory of Xin'an Medicine, Anhui University of Chinese Medicine, Ministry of Education, Hefei, China; ^5^Anhui Province Key Laboratory of Pharmaceutical Technology and Application, Anhui University of Chinese Medicine, Hefei, China; ^6^Engineering Technology Research Center of Modernized Pharmaceutics, Education Office of Anhui Province, Hefei, China

## Abstract

*Panax notoginseng* (PN) has become the most widely used dietary supplement and herbal in Asian countries. The effect of micronization on PN is not entirely clear. The aim of this study was to investigate the effects of particle size of *Panax notoginseng* powder (PNP) and the potential to improve the bioavailability. The results showed that particle size reduction significantly changed the *Panax notoginseng* saponins (PNS) *in vitro* dissolution and *in vivo* pharmacokinetics. The size of the *Panax notoginseng* powder (PNP) ranges from 60 to 214 *μ*m. The surface morphology and thermal properties of PNP were extensively characterized, and these changes in physicochemical properties of PNP provide a better understanding of the *in vitro* and *in vivo* release behaviors of PNS. The *in vitro* studies demonstrated that the dissolution of PNS and particle size were nonlinear (dose- and size-dependent). The pharmacokinetics parameters of PNP in rats were determined by UHPLC-MS/MS. Powder 4 (90.38 ± 8.28 *μ*m) showed significantly higher AUC_0-T_ values in plasma (*P* < 0.05). In addition, we also investigated the influence of the hydrothermal treatment of PNP. The results showed that the PNS *in vitro* release and *in vivo* bioavailability of PNP pretreatment at 40°C were the highest. This suggests that PNP with a particle size of around 90 *μ*m and heat pretreatment at 40°C would be beneficial. These results provided an experimental basis, and it was beneficial to choose an appropriate particle size and hydrothermal temperature when PNP was used in clinical treatment.

## 1. Introduction

*Panax notoginseng* (PN), the dry root and rhizome of *Panax notoginseng* (Burk.) F. H. Chen, has been a well-known traditional Chinese medicine (TCM) and has a long history in China [1]. PN has definite effects of dispersing blood stasis, hemostasis, detumescence, and analgesia [2]. *Panax notoginseng* saponin (PNS) is one of the key bioactive components of PN [3]. Several studies have shown that PNS possesses various pharmacological activities, such as being antithrombotic, neuroprotective, anti-inflammatory, and hyperlipidemia [4–7]. Besides the medicinal use, PN is also used extensively as a functional food in China [8]. PN and PN-based products were listed as an item that can be used as a dietary supplement by the US Dietary Supplement Health and Education Act of 1994 [9–11].

**Figure 1:**
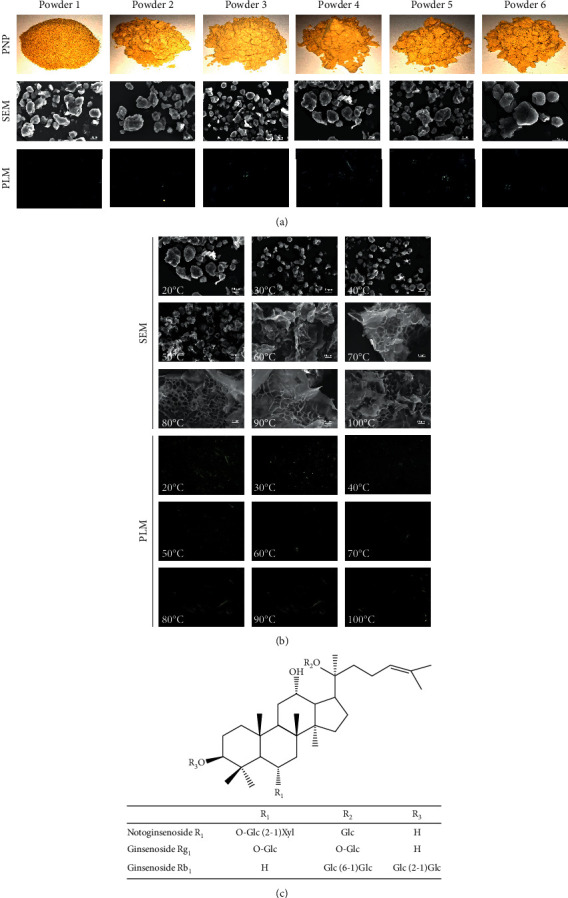
(a) Morphological appearance and PLM images of the PNP with different particle sizes, (b) SEM and PLM images of the hydrothermally treated PNP, and (c) structure of notoginsenoside R_1_ and ginsenosides Rg_1_ and Rb_1_.

*Panax notoginseng* powder (PNP) is the crushed product of the dry roots of PN because it was convenient to take and has obvious healthcare effects; it was favored by people with cardiovascular and cerebrovascular diseases [12, 13]. Substantial studies have demonstrated that the dissolution of the main active ingredients of TCM and its oral bioavailability have often been related to the degree of crushing [14–16]. Generally, the smaller particle size is favorable for the dissolution of active ingredients and improving clinical efficacy [17, 18]. However, there is no uniform-sized PNP in the market. Studies have shown that there were significant differences in the dissolution of PN coarse powder, fine powder, and micropowder, but micropowder was not better than fine powder in terms of dissolution [19, 20]. However, in these studies, the particle size of PNP was not divided in detail. In addition, studies found that some TCM containing more starch granules, such as *Fritillariae bulbus*, would be gelatinized and form a dense layer with the increase of solvent temperature and the participation of water, making it difficult for water molecules to enter the inside of the medicine, making the effective ingredients difficult to dissolution [21, 22]. At present, there are few studies on the interaction between hydrothermal temperature and PNP. In order to systematically investigate the effect of particle size on the dissolution of PNP *in vitro*, and the correlation between *in vitro* dissolution and *in vivo* bioavailability, six types of PNP (from 60 to 214 *μ*m) were prepared in this study. The properties, *in vitro* dissolution, and *in vivo* bioavailability of PNP with different particle sizes were investigated, and the effect of the water temperature of the suspension of PNP on its dissolution and absorption was also investigated, so as to provide a reference for the production specification and usage methods of PNP. We firstly assessed the effects of particle size and hydrothermal treatments on the morphological properties and thermal properties of PNP, and the *in vitro* dissolution studies were performed in pure water and simulated gastric fluid, and the pharmacokinetics of PNP with different particle sizes and different hydrothermal temperature treatments were performed in rats. Moreover, notoginsenoside R_1_ and ginsenosides Rg_1_ and Rb_1_ (the chemical structures of which are shown in Figure 1(c)) are considered to be the main components of PNS and were determined in the dissolution and pharmacokinetics studies [23, 24]. The aim of this study was to provide further information to establish guidelines for the use of PNP.

## 2. Materials and Methods

### 2.1. Chemicals and Materials

The *P. notoginseng* root was purchased from Anhui Boyao Qiancao Traditional Chinese Medicine Co. Ltd. (Anhui, China) and authenticated by Professor Peng Huasheng (Anhui University of Traditional Chinese Medicine). Pepsin from porcine gastric mucosa was supplied by Yuanye Co. Ltd. (Shanghai, China). The water used was distilled and passed through a Milli-Q water purification system (Millipore, Bedford, USA). All other reagents were of analytical grade or pharmaceutical grade.

### 2.2. Animals

All Sprague Dawley (SD) rats (weight, 250 ± 20 g) were purchased from the Experimental Animal Center of Jinan Pengyue Co. Ltd. (Jinan, China). All animal welfare and experimental procedures were approved by the Animal Ethical Committee of Anhui University of Chinese Medicine (Approval no. 1107261911002543) and were in accordance with the Guide for the Care and Use of Laboratory Animals. The rats were acclimated for approximately 7 d with a 12 h light and dark cycle throughout the experiment. The rats were housed and given free access to food and water, and the temperature was maintained at room temperature (25°C).

### 2.3. PNP Preparation

The PNP was crushed by a highly efficient pulverizer, and the particle size was analyzed using a laser particle size analyzer (BT-9300HT, BAITE, Dandong, China) using water as the dispersion medium.

### 2.4. Characterization of PNP

#### 2.4.1. Morphological Appearance

The morphological appearance of PNP was observed by scanning electron microscopy (SEM, S3400N HITACHI) [25]. The hydrothermally treated samples (Powder 4) were soaked in water for 5 min at 20, 30, 40, 50, 60, 70, 80, 90, or 100°C, respectively, and then the PNP was prepared by freeze drying. The powders were placed on double-sided adhesive tape on SEM stubs and coated with gold [26, 27].

#### 2.4.2. Polarized Light Microscopy (PLM)

The prepared PNP was characterized by polarization microscope (CK-500, Caikon, China) at room temperature [21, 25]. 500 mg of PNP in a tube was diluted with distilled water (2 mL). The hydrothermally treated samples (Powder 4) were steeped in water for 5 min at 20, 30, 40, 50, 60, 70, 80, 90, or 100°C, respectively.

#### 2.4.3. Differential Scanning Calorimetry (DSC)

DSC analysis of the PNP was measured using differential scanning calorimetry (DSC200F3, NETZSCH, Germany) under an ultrapure nitrogen atmosphere [28, 29]. The PNP (3 mg) was mixed with 12 *μ*L of distilled water, hermetically sealed in an aluminum pan, and equilibrated at 4°C for 24 h. The scanning temperature was then increased from 20°C to 220°C at a heating rate of 10°C/min [30, 31].

### 2.5. Analysis of Notoginsenoside R_1_ and Ginsenosides Rg_1_ and Rb_1_ by HPLC

The content of notoginsenoside R_1_ and ginsenosides Rg_1_ and Rb_1_ in the samples was determined using HPLC and the method reported in the Chinese Pharmacopoeia (2015). HPLC analyses were performed on an Ultimate 3000 series system (Thermo Fisher Scientific, China) consisting of a quaternary pump, DAD detector, and autosampler, and the data were analyzed using Chromeleon 7 software. A Thermo Syncronis C_18_ column (250 × 4.6 mm, 5.0 *µ*m) was adopted for the separation. The mobile phase consisted of A (pure water) and B (acetonitrile). The gradient mode was as follows: 0–12 min, 19% A; 12–60 min, 19% ∼ 36% A. The flow rate was 1.0 mL/min, and the detection wavelength was set to 203 nm. The column temperature was 25°C, and the sample injection volume was set to 20 *µ*L.

### 2.6*. In Vitro* Dissolution Studies

The effects of particle size and hydrothermal treatment (Powder 4 was treated at 30, 40, 50, and 100°C) on the dissolution behavior of the PNP were investigated. Dissolution studies were carried out on a dissolution apparatus (ZRS-8L, Tianda Tianfa, China) with a rotation speed of 75 rpm according to the China Pharmacopoeia apparatus III paddle method [32, 33]. The tests were performed in 150 mL of freshly prepared aqueous solution and simulated gastric fluid (10 g/L pepsin, 0.1 M HCL, pH = 1.2, ChP 2015). Maintaining a temperature of 37 ± 0.5°C, samples of 4 mL each were withdrawn and replaced with fresh medium of equal volume at fixed time points of 0, 5, 10, 15, 20, 25, 30, 60, 90, and 180 min, respectively. The content of notoginsenoside R_1_ and ginsenosides Rg_1_ and Rb_1_ in the medium was determined by HPLC as described above. Because the dissolution of ingredients was affected by the particle size, the dissolution rate calculated using the revised equation is as follows:

(1) $$\begin{array}{c} \text{dissolution}\%=\frac{Ve\sum_{1}^{n-1}Ci+V0Cn}{m_{\text{PNP}}}\times 100\%, \end{array}$$

where *V*_*e*_ means 4 mL, *C*_*i*_ means the sample concentration of release medium from the *i*th permutation, *V*_0_ means the total volume of release medium, *n* means the number of replacement release medium, and *m*_PNP_ means the weight of PNP.

### 2.7. *In Vivo* Pharmacokinetic Study

Before administration, sixty rats were fasted for 12 h, but given free access to water. The rats were randomly divided into ten groups, six rats in each group. Rats were pretreated with different particle sizes of PNP (Powders 1 ∼ 6, 540 mg/kg, i.g.) or the PNP (Powder 4) treated at different temperatures (30, 40, 50, and 100°C, 540 mg/kg, i.g.). After intragastric administration of PNP, 200 *µ*L of blood was collected into heparinized tubes, at 0.08, 0.17, 0.25, 0.5, 0.75, 1, 1.5, 2, 4, 6, 12, 24, and 48 h from the ophthalmic veins and centrifuged at 3000 rpm for 10 min. Plasma was separated and frozen at −20°C for analysis. 10 *μ*L of digoxin (1.0 *μ*g/mL) as an internal standard (IS) was added to each blood sample (100 *μ*L), which was pretreated according to the previously published method [34], and the concentrations of notoginsenosides were analyzed using a validated UHPLC-MS/MS method.

The notoginsenosides in the biological samples were analyzed using a validated UHPLC-MS/MS method. The UHPLC-MS/MS system (UHPLC-MS/MS-5500 system, AB Sciex Instrument, USA) contained an ExionLC AD UHPLC system and a QTRAP 5500 triple quadrupole mass spectrometry instruments equipped with an electron spray ionization (ESI) source. The data acquisition and analysis were performed using MultiQuant software (AB Sciex Instrument). Liquid chromatographic separation was carried out at 25°C using a 1.8 *µ*m, 100 × 2.1 mm Epic C18 column. The mobile phase was composed of acetonitrile (solvent A) and water containing 0.1 mM ammonium chloride (solvent B). An optimized gradient elution condition was set as 10% A (0 ∼ 0.02 min), 10% ∼ 75% A (0.02 ∼ 5 min), 75% A (5 ∼ 5.5 min), 75% ∼ 10% A (5.5 ∼ 6 min), and 10% A (6 ∼ 9 min). A sample volume of 2 *µ*L was injected, and a flow rate of 0.2 mL/min was employed. The ESI source was operated in negative ionization mode, and the MS conditions were set as follows: desolvation temperature, 250°C; heat block temperature, 550°C; nebulizer gas flow, 3 L/min; drying gas flow, 15 L/min; and interface voltage, 3.5 kV. Data were acquired by multiple reaction monitoring (MRM).  Table 1 lists the quantitatively optimized parameters, including the declustering potential (DP) and collision energy (CE).

### 2.8. Statistical Analysis

All data are expressed as the mean ± SD. Differences between groups were compared by SPSS Statistics 24 using the independent-samples *t*-test or paired-samples *t*-test. Statistically significant differences were determined at *P* < 0.05 and *P* < 0.01.

## 3. Results and Discussion

### 3.1. Particle Size and Morphological Properties of PNP

The particle size measurements in Table 2 and the size distribution of the PNP varied significantly. The morphology of different particle sizes and hydrothermally treated PNP was observed by naked eyes and SEM and under polarized light (Figure 1(a)). The PNP appeared in grayish yellow and with the particle size decreased. The PNP tended to become disrupted and aggregate. The PNP had the birefringence characteristic (Maltese cross) of the starch granules when viewed using polarized light, and as the particle size of the PNP decreased, the Maltese cross of the starch granules became increasingly obvious. During heating, as shown in Figure 1(b), at temperatures above 50°C, the birefringence gradually disappeared. Micromorphological characteristics of the PNP and heat-treated PNP at 20 to 100°C were further observed using SEM. During heating, the PNP maintained a granular morphology until the temperature reached 50°C; above this temperature, the PNP gradually absorbed water, expanded in the radial direction, and formed a cross-linked structure. Eventually, all the PNP melted and fused into larger aggregates.

### 3.2. Thermal Properties

The gelatinization of PNP is important in active ingredient release, and DSC is the most accepted way to measure gelatinization temperatures [28–30, 35, 36]. The gelatinization temperatures of PNP were 40.03 ∼ 46.87°C, 113.41 ∼ 115.77°C, and 121.12 ∼ 122.55°C for the onset temperature of gelatinization (*T*_o_), the peak temperature (*T*_p_), and the conclusion temperature (*T*_c_), respectively (Table 3). These results indicate that the onset temperature of gelatinization of PNP was in the range of 40 ∼ 50°C, and the gelatinization property could be independent of granule size.

### 3.3. *In Vitro* Dissolution Study

**Figure 2:**
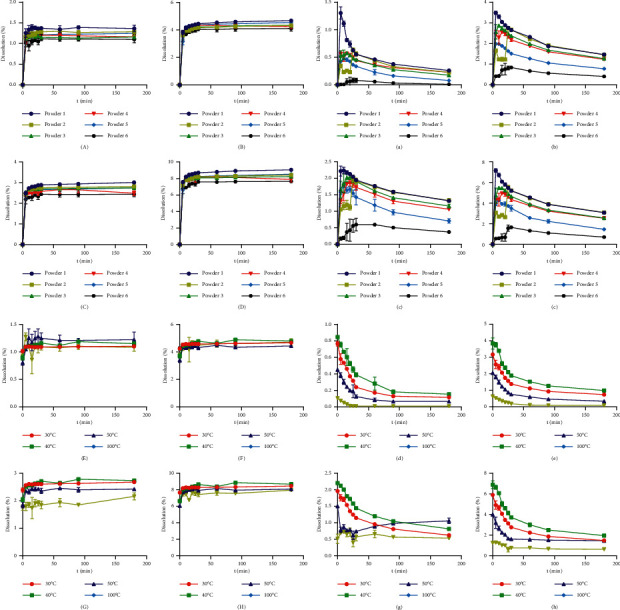
Dissolution profiles of the PNP in pure water (A ∼ H) and simulated gastric fluid (a ∼ h). A, E, a, e: notoginsenoside R_1_; B, F, b, f: ginsenosides Rg_1_; C, G, c, g: ginsenosides Rb_1_; D, H, d, h: PNS. 1 ∼ 6 correspond to Powders 1 ∼ 6, and 30, 40, 50, and 100°C correspond to the PNP treated at different hydrothermal temperatures.

The effects of particle size and temperature on saponin dissolution are shown in Figure 2. The tests were carried out in aqueous solution (Figure 2) and simulated gastric fluid (Figure 2). The notoginsenoside R_1_ and ginsenosides Rg_1_ and Rb_1_ released from the PNP with different particle sizes (Powders 1 ∼ 6) and the hydrothermally treated PNP at different temperatures (30, 40, 50, and 100°C) were comparable. From Figure 2, the PNP showed a high dissolution rate with >90% of the saponins released within 30 min. The dissolution of the saponins from Powder 1 was higher than that of the other powders, while the dissolution of the saponins of Powder 6 was the lowest. The dissolution results showed that the dissolution behavior of notoginsenoside R_1_ and ginsenosides Rg_1_ and Rb_1_ could be independent of particle size. As the particle size decreased to a certain extent (such as in Powder 6), the saponins also decreased (the PNS decreased from 90.51 to 76.51 mg/g compared with Powder 1), and the peak of the PNS from Powder 1 and Powder 6 was statistically significant (*P* < 0.05). The effects of temperature are shown in Figure 2. The amount of saponins contained in the PNP treated at 40°C was higher than that at other temperatures. Combined with the SEM and DSC results, it can be inferred that the starch granules from the PNP treated at 30°C and 40°C were in the gelatinization process of water absorption [21]. At this stage, the water molecules enter the starch granule gap in the PNP, a small amount of water is absorbed by the starch granules, and the swelling makes the active ingredients in the PNP more soluble. Upon heating and entering the gelatinization temperature, the samples enter the irreversible water absorption stage, and so the PNP treated at 50°C and 100°C was at this stage, and a large amount of water was quickly absorbed by the starch granules, causing the granules to swell to 600% ∼ 1000% of their original volume [37]. Meanwhile, the swollen PNP aggregated and formed a cross-linked structure (Figure 1(b)), resulting in the active ingredient being difficult to dissolve. In the daily use of PNP, the maximum temperature of water consumption is 100°C, so further increasing temperatures were not studied.

From Figure 2, Powder 1 (released for 180 min) showed the sustained release of notoginsenoside R_1_ and ginsenosides Rg_1_ and Rb_1_ with a statistically significant difference (*P* < 0.01) compared to Powder 6. Notoginsenoside R_1_ and ginsenosides Rg_1_ and Rb_1_ are extremely unstable in simulated gastric fluid, which was in agreement with results previously reported [38–40]. The maximum dissolution of PNP with different particle sizes in simulated gastric fluid was lower than that when water was used as the release medium, but the peak time in simulated gastric fluid was earlier than that of water. The dissolution of the saponins contained in the PNP at 40°C was higher than that at other temperatures (Figure 2). The maximum dissolution of notoginsenoside R_1_ was 1.11-, 1.88-, and 5.28-fold greater than that of the other temperature groups (30, 50, and 100°C, respectively). Ginsenoside Rg_1_ was 1.21-, 1.87-, and 6.09-fold greater than that of the other temperature groups, and ginsenoside Rb_1_ was 1.12-, 1.47-, and 3.06-fold greater than that of the other temperature groups, respectively. From Figure 2, the dissolution of PNP showed a high dissolution rate with >90% of the saponins released within 10 min, which may be because the degradation rate of the saponins in the gastric juice environment is higher than the dissolution rate.

### 3.4. *In Vivo* Study

**Figure 3:**
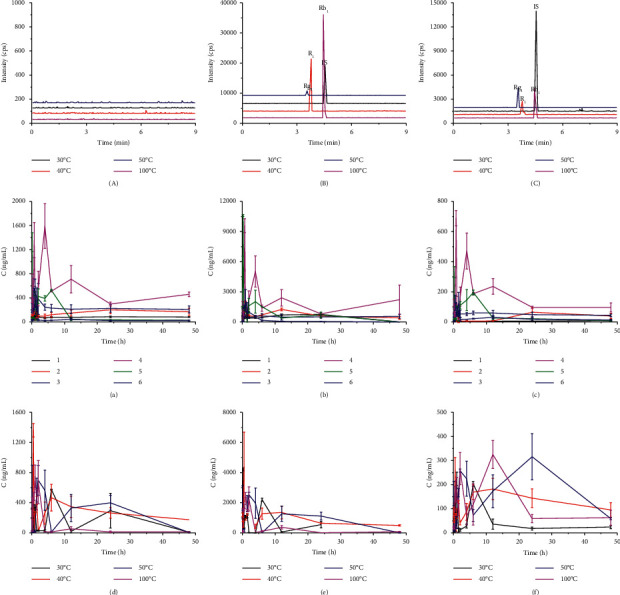
MRM chromatograms and the plasma concentration-time profiles of notoginsenoside R_1_, Rg_1_, and Rb_1_ in rats following intragastric administration of different sizes of PNP at a dose of 540 mg/kg. A: blank rat plasma, B: blank plasma spiked with notoginsenosides and IS, C: rat plasma collected at 30 min after intragastric administration of 540 mg/kg PNP. a, d: notoginsenoside R_1_; b, e: ginsenosides Rg_1_; c, f: ginsenosides Rb_1_. Samples 1 ∼ 6 correspond to Powders 1 ∼ 6, and 30, 40, 50, and 100°C correspond to the PNP treated at different hydrothermal temperatures.

The UHPLC-MS/MS methods were subjected to validation based on specificity, linearity, sensitivity, intraday and interday precision, accuracy, recovery, matrix effect, and stability. The specificity was analyzed by comparing chromatograms of blank plasma (from 6 different rats), blank plasma with IS, notoginsenoside R_1_, ginsenosides Rg_1_ and Rb_1_, and plasma samples after intragastric administration of PNP. As shown in Figure 3, there was no significant endogenous peak interference within the retention time of the IS, notoginsenoside R_1_, or ginsenosides Rg_1_ and Rb_1_. The linearity of the calibration curves was found to be high in the range of 0.9996 ∼ 499.8 ng/mL for notoginsenoside R_1_ (*r* = 0.9991), 0.9744 ∼ 487.2 ng/mL for ginsenoside Rg_1_ (*r* = 0.9947), and 1.0843 ∼ 541.8 ng/mL for ginsenoside Rb_1_ (*r* = 0.9991). The intra- and interday precisions of notoginsenoside R_1_ and ginsenosides Rg_1_ and Rb_1_ in rat plasma were less than 15% in terms of RSD%, and the accuracy was below 20% in terms of RE%. The recovery value for notoginsenoside R_1_ was 83.67% at 4.998 ng/mL, 84.44% at 49.98 ng/mL, and 93.56% at 199.9 ng/mL; for ginsenoside Rg_1_, the recoveries were 76.86% at 4.887 ng/mL, 88.64% at 48.72 ng/mL, and 85.57% at 194.9 ng/mL; for ginsenoside Rb_1_, the recoveries were 73.64% at 5.468 ng/mL, 91.05% at 54.18 ng/mL, and 82.66% at 216.7 ng/mL; and the standard deviations (SD) were all lower than 7.23%. This method was suggested to be high throughput and reliable.

The mean plasma concentration-time curves after oral administration of PNP of different sizes and hydrothermally treated treatments are shown in Figure 3. The pharmacokinetics parameters are listed in Tables 4 and 5. From Figure 3 and Table 4, the results show that the AUC_0-T_ of the three saponins in Powder 4 was significantly higher than that in the other powders (*P* < 0.05), and the AUC_0-T_ of notoginsenoside R_1_ was 6.21-fold greater than that of Powder 1 and 13.31-fold greater than that of Powder 6. The AUC_0-T_ of ginsenoside Rg_1_ in Powder 4 was 5.69-fold and 15.69-fold that of Powder 1 and Powder 6, respectively. Finally, the AUC_0-T_ of ginsenoside Rb_1_ in Powder 4 was 6.2-fold and 13.3-fold that of Powder 1 and Powder 6, respectively. The results showed that, after intragastric administration of Powder 4, the concentrations of notoginsenoside R_1_ and ginsenosides Rg_1_ and Rb_1_ in systemic circulation were higher than those of the other powders. The *T*_max_ values of the three saponins from Powder 5 were much smaller than those of the other powders, and the C_max_ of ginsenoside Rg_1_ was higher than that of other powders, indicating that, after the administration of Powder 5, the saponins were absorbed quickly in the body and reach a peak rapidly. After Powders 4 and 5 were administered by intragastric administration, notoginsenoside R_1_ and ginsenosides Rg_1_ and Rb_1_ exhibited double peaks in the C-T figures (Powder 4 was more obvious), which were consistent with results previously reported [21, 41]. This result could be due to differential rates of absorption along the gastrointestinal tract, and this prolonged residence time of the drug in the body could be beneficial to the maintenance of the drug effect, but the mechanisms of the double peaks remain to be determined [42, 43]. The data suggested that the decrease in particle size can successfully improve the bioavailability within a range of 213.93 ∼ 90.38 *μ*m (Powders 1 ∼ 4), and the result was not in agreement with the dissolution data (Figure 2). This phenomenon could be related to the high concentration of ginsenosides having a tendency to form self-micelles that might prevent them from permeation and absorption [44]. Figure 3 and Table 5 show that the AUC_0-∞_ of notoginsenoside R_1_ and ginsenoside Rg_1_ after the administration of the PNP treated at 40°Cwas 3.33-, 1.95-, and 7.59-fold and 3.27-, 1.45-, and 5.59-fold greater than that at 30, 50, and 100°C, respectively. The AUC_0-∞_ of ginsenoside Rb1 after the administration of PNP at 50°C was 9.29-, 1.68-, and 2.47-fold greater than that at 30, 40, and 100°C, respectively.

By comparing the data of PNS *in vivo* and *in vitro*, we could find that the group of different particle size *in vivo* bioavailability result was inconsistent with *in vitro* dissolution. This indicated that within the range of 213.93 ∼ 90.38 *μ*m (Powders 1 ∼ 4), the oral bioavailability was higher as the particle size decreases, but when the particle size decreases to a critical value (90 *μ*m), it would affect the absorption of PNS. The group of hydrothermal temperatures *in vivo* result was in good agreement with the *in vitro* dissolution data that the higher dissolution *in vitro* led to faster absorption *in vivo*. It could be concluded that temperature may affect the absorption of PNS, and the highest bioavailability of PNP was after treatment at 40°C.

## 4. Conclusion

This work confirmed that the micronization of PN and hydrothermal treatment had a significant effect on the properties and *in vitro* and *in vivo* behaviors of PNP. *In vitro* studies have shown that the amount and rate of dissolution of saponins are independent of the particle size in the range of 213.93 to 59.89 *μ*m, but the hydrothermal PNP at different temperatures could affect the dissolution of saponins (40°C was the most effective). The bioavailability of Powder 4 (90.38 ± 8.28 *μ*m) was better than that of the other particle size powders, and it was found that Powder 4 was the easiest to prepare during the preparation process. The bioavailability of PNP after treatment at 40°C was the highest. In summary, it is recommended to use PNP with a particle size around 90 *μ*m and suspend in 40°C water. This work deeply explores the influence of micronization on PN and provides a reference for follow-up development of other TCM to add further insights into the effects of micronization.

## Figures and Tables

**Table 1:** MRM parameters of notoginsenosides and internal standard.

Analyte	Precursor (*m*/*z*)	Product (*m*/*z*)	CE (eV)	DP (eV)
R_1_	967.7	637.5	−22	−46
Rg_1_	835.7	637.4	−138	−55
Rb_1_	1143.7	1107.7	−294	−59
IS	815.5	779.4	−82	−35

**Table 2:** The particle sizes of PNP (*n* = 3).

Sample	Particle size (*μ*m)
Powder 1	213.93 ± 7.06
Powder 2	170.02 ± 2.72
Powder 3	103.77 ± 2.78
Powder 4	90.38 ± 8.28
Powder 5	72.77 ± 0.13
Powder 6	59.89 ± 0.06

**Table 3:** Thermal parameters of the PNP (*n* = 3).

Sample	*T*_o_ (°C)	*T*_p_ (°C)	*T*_c_ (°C)
Powder 1	46.87 ± 5.50	114.70 ± 1.50	122.14 ± 0.79
Powder 2	45.38 ± 4.36	115.66 ± 1.53	122.29 ± 2.28
Powder 3	42.02 ± 4.82	115.07 ± 3.64	122.55 ± 3.05
Powder 4	40.03 ± 3.59	115.70 ± 1.51	122.34 ± 2.27
Powder 5	45.33 ± 2.02	113.41 ± 4.93	121.12 ± 2.80
Powder 6	40.74 ± 4.05	115.77 ± 1.55	122.50 ± 1.40

**Table 4:** The pharmacokinetic parameters of notoginsenosides following the intragastric administration of 540 mg/kg PNP (*n* = 6)

Notoginsenoside	Sample	*C*_max_ (ng/mL)	*T*_max_ (h)	*t*_1/2_ (h)	MRT (h)	AUC_0-T_ (ng·h/mL)	AUC_0-∞_ (ng·h/mL)
R_1_	1	96.77 ± 8.33	0.75 ± 0.43	61.40 ± 24.40	24.39 ± 0.28 ^*∗∗*^	3927.74 ± 168.19 ^*∗∗*^	15934.90 ± 3756.68
	2	214.85 ± 46.16	18.67 ± 8.64	30.85 ± 17.86	25.56 ± 1.18 ^*∗∗*^	7988.59 ± 1539.43 ^*∗∗*^	22347.20 ± 3962.25 ^*∗*^
	3	480.12 ± 184.33	1.31 ± 0.91	45.18 ± 19.94	23.28 ± 1.62 ^*∗∗*^	10703.32 ± 195.88 ^*∗∗*^	26067.99 ± 2653.31 ^*∗∗*^
	4	2520.17 ± 699.78	1.83 ± 1.68	10.83 ± 5.83 ^*∗∗*^	20.42 ± 0.76 ^*∗∗*^	24392.12 ± 1209.31 ^*∗∗*^	25608.71 ± 963.71 ^*∗∗*^
	5	1325 ± 70.20	0.15 ± 0.03	11.01 ± 0.13 ^*∗*^	10.58 ± 0.19 ^*∗∗*^	5136.15 ± 139.26 ^*∗∗*^	6490.01 ± 2116.24
	6	585.16 ± 113.86	0.92 ± 0.30	58.53 ± 29.21	16.55 ± 1.66	1832.64 ± 147.30	4246.85 ± 1457.93

Rg_1_	1	1078.91 ± 221.73	0.43 ± 0.15	22.28 ± 17.71	13.64 ± 1.25 ^*∗∗*^	14876.10 ± 3408.79	44835.03 ± 14993.19
	2	1997.5 ± 325.40	0.96 ± 0.10	17.02 ± 3.81	19.36 ± 2.01 ^*∗∗*^	33292.35 ± 6663.89 ^*∗∗*^	58485.02 ± 38882.91 ^*∗*^
	3	1577.33 ± 8.17	1.17 ± 0.41	57.43 ± 24.07 ^*∗∗*^	23.30 ± 2.23 ^*∗∗*^	26219.02 ± 2116.79 ^*∗∗*^	73657.82 ± 31451.60 ^*∗∗*^
	4	10134.52 ± 4812.27	1.83 ± 1.68	7.72 ± 0.83 ^*∗∗*^	23.04 ± 1.70 ^*∗∗*^	84696.89 ± 13040.28 ^*∗∗*^	85766.94 ± 12948.66 ^*∗∗*^
	5	10590 ± 230.56	0.29 ± 0.10 ^*∗∗*^	3.52 ± 0.72	8.72 ± 0.38 ^*∗∗*^	23225.07 ± 2017.82 ^*∗∗*^	23433.90 ± 1935.81
	6	2270.72 ± 670.85	1.04 ± 0.25	5.78 ± 1.29	4.7 ± 0.36	5396.88 ± 349.55	5763.17 ± 335.36

Rb_1_	1	9.84 ± 4.99 ^*∗*^	36 ± 13.14 ^*∗∗*^	42.00 ± 24.07	28.69 ± 3.80 ^*∗∗*^	296.79 ± 85.85 ^*∗∗*^	770.01 ± 225.94
	2	64.52 ± 27.33 ^*∗*^	22.00 ± 4.90	11.17 ± 5.32	29.65 ± 0.73 ^*∗∗*^	1756.56 ± 634.27 ^*∗∗*^	2115.94 ± 361.33
	3	76.90 ± 9.97	18.00 ± 8.49	83.46 ± 52.21	22.57 ± 1.00 ^*∗∗*^	2444.21 ± 100.46 ^*∗∗*^	7518.63 ± 3769.97
	4	741.18 ± 408.39	2.05 ± 1.78	12.37 ± 7.81 ^*∗*^	18.33 ± 1.17	7212.91 ± 169.41 ^*∗∗*^	10109.07 ± 4381.42
	5	331.05 ± 16.35	0.15 ± 0.03	10.85 ± 2.52 ^*∗*^	11.50 ± 0.32 ^*∗∗*^	2031.16 ± 61.08 ^*∗∗*^	2160.99 ± 71.61
	6	140.71 ± 21.88	0.79 ± 0.37	56.67 ± 31.54	18.56 ± 1.29	1068.46 ± 179.46	2388.17 ± 841.41

Samples 1 ∼ 6 correspond to Powders 1 ∼ 6. ^*∗*^*P* < 0.05,  ^*∗∗*^*P* < 0.01 versus Powder 6.

**Table 5:** The pharmacokinetic parameters of notoginsenosides following 540 mg/kg intragastric administration of PNP treated at different temperatures (*n* = 6)

Notoginsenoside	Sample (°C)	*C*_max_ (ng/mL)	*T*_max_ (h)	*t*_1/2_ (h)	MRT (h)	AUC_0-T_ (ng h/mL)	AUC_0-∞_ (ng h/mL)
R_1_	30	572.15 ± 3.00	5.67 ± 0.82	7.99 ± 3.92	17.77 ± 1.70 ^*∗*^	7580.39 ± 1242.09	7716.85 ± 1259.98
	40	1390.85 ± 652.91 ^*∗*^	0.54 ± 0.10	49.37 ± 22.02	20.35 ± 1.27 ^*∗∗*^	13164.57 ± 1381.68 ^*∗∗*^	25703.83 ± 5377.46 ^*∗∗*^
	50	796.67 ± 98.29	1.93 ± 1.26	6.21 ± 0.32 ^*∗*^	17.27 ± 0.75	12599.54 ± 1698.24 ^*∗∗*^	13159.87 ± 1263.91
	100	824.20 ± 86.07	1.33 ± 0.52	42.03 ± 27.99	8.62 ± 0.65	2580.91 ± 378.24	3388.29 ± 951.55

Rg_1_	30	4223.25 ± 5.32 ^*∗*^	0.23 ± 0.05 ^*∗*^	4.31 ± 0.54	9.37 ± 1.38	14825.41 ± 1107.87	17920.93 ± 2148.99
	40	4469.58 ± 2181.84	0.46 ± 0.10	30.04 ± 13.17	18.75 ± 0.97 ^*∗∗*^	39965.99 ± 4336.01 ^*∗∗*^	58687.93 ± 12288.43 ^*∗∗*^
	50	2982.75 ± 610.15	2.01 ± 1.24	4.51 ± 0.44	15.81 ± 0.96 ^*∗∗*^	40506.20 ± 6170.47 ^*∗∗*^	40542.23 ± 6159.83 ^*∗*^
	100	2665.42 ± 378.99	1.33 ± 0.52	7.13 ± 4.56	9.51 ± 0.84	10354.88 ± 1599.79	10495.51 ± 1490.15

Rb_1_	30	245.16 ± 62.15	1.17 ± 0.41	8.51 ± 7.08 ^*∗*^	15.60 ± 2.63	1951.25 ± 358.41 ^*∗∗*^	2619.71 ± 1138.15
	40	295.87 ± 82.18	0.54 ± 0.10 ^*∗∗*^	36.26 ± 5.61	22.05 ± 1.09 ^*∗∗*^	6462.73 ± 830.14	14491.60 ± 7236.11
	50	381.11 ± 158.24	2.67 ± 1.03	21.15 ± 13.78	21.15 ± 1.29	9138.96 ± 3314.69 ^*∗*^	24329.15 ± 14404.06
	100	345.88 ± 51.91	10.17 ± 4.49	35.85 ± 15.84	14.49 ± 6.22	6103.76 ± 328.86	9846.49 ± 1855.47

Sample 30, 40, 50, and 100°C correspond to the PNP treated at different hydrothermal temperatures.  ^*∗*^*P* < 0.05,  ^*∗∗*^*P* < 0.01, versus 100°C.

## Data Availability

The data used to support the findings of this study are available from the corresponding author upon request.
